# Supplementary material for: Temporal kinetics of brain state effects on visual perception
Source: Sci Rep. 2026 May 9;16:14689. doi: 10.1038/s41598-026-50974-5 (PMC13157504; doi:10.1038/s41598-026-50974-5)
Supplement: Supplementary file 1 — Supplementary Material 1 [file 41598_2026_50974_MOESM1_ESM.docx]

***Supplementary Material***

***S1-Transition based on reported labels***

Here we test for transition based on actual reports over time. To achieve this, we calculated the frequency of ON (labels 4 & 5) and OFF (labels 1 & 2) states for each participant across all FQ+N trials, resulting in 25 ON and OFF values per participant. These values reflect changes in attention and inattention over time with a resolution of ~2 seconds. In the left figure, we show the averaged absolute frequencies over time relative to FQ. Next, we tested how ON and OFF ratings changed over time by correlating trial number (1–25) with ratings using Spearman’s correlation, separately for ON and OFF. The resulting correlation coefficients were tested against zero using a one-sample *t*-test. ON ratings decreased over time (*t*_19_ = -2.2, *r*_mean_ = -0.15, *P* = .03), while OFF ratings increased (*t*_19_ = 4.05, *r*_mean_ = 0.14, *P* = .0007), as shown in the right figure. Black dots represent individual Spearman correlation coefficients for OFF changes over time, and red dots represent those for ON. Additionally, we compared the mean correlation coefficients to a surrogate distribution generated under the assumption of no systematic change over time. We pseudorandomly shuffled trial labels, reassigned them across the 25 FQ+N trials, computed correlation coefficients per participant, and averaged them. This process was repeated 1000 times to create a distribution of 1000 mean correlation values. Finally, we determined the probability of the observed ON and OFF label changes within this distribution. The observed correlation values exceeded the confidence intervals of the surrogate distribution (two-sided 95% CI: [-0.072, 0.073]; *P*_ON_ = .00003, *P*_OFF_ = .0013).


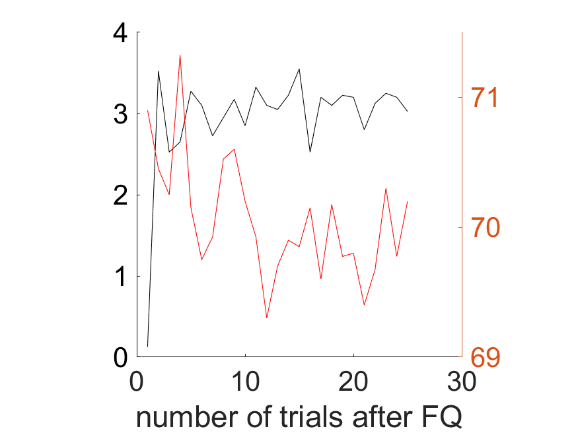

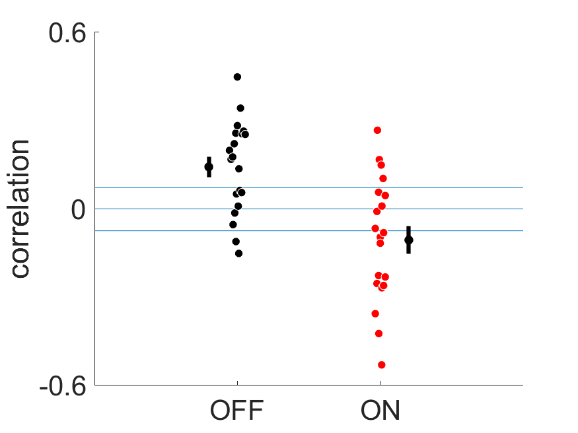


***Figure S1***. Transition of brain states over time. Frequency of predicted ON (red) and OFF (black) trials across trials following a thought probe. These values reflect changes in attention and inattention over time with a resolution of ~ 2 seconds since a trial on average lasted 1.90 sec (left). Correlation of trial numbers and participants’ individual brain state ratings to test how ON (red) and OFF (black) ratings changed over time. ON ratings decreased, while OFF ratings increased over time. Mean ON and OFF ratings exceeded the confidence intervals (blue lines) of a surrogate distribution generated under the assumption of no systematic change over time (right).

***S2-Transition based on absolute values***

Additionally, we conducted an analysis in which we calculated transition times based on the absolute frequencies of observed labels. While participant reports are most informative, the number of thought probes was limited. These were pseudorandomly distributed with at least two intervening trials, but not all participants experienced every possible interval (2–25 trials), meaning mind-wandering ratings were not available for every FQ+N. To analyze the temporal evolution of mind-wandering, we identified FQs where participants responded ON-task, then grouped subsequent thought probe ratings by their temporal distance from these reports and averaged them. This provided mean ratings for each participant based on trial intervals (N trials) after an ON report. We assessed how ON and OFF ratings changed over time by correlating available trial intervals with ratings using Spearman’s correlation, separately for ON and OFF. Given the low number of ratings per participant, available intervals varied. The resulting correlation coefficients were tested against zero using a one-sample t-test. ON ratings decreased over time (*t*_19_ = -3.89, *r*_mean_ = -0.27, *P* = .00096), while OFF ratings increased (*t*_19_ = 4.09, *r*_mean_ = 0.28, *P* = .0006). Figure S2 shows individual Spearman correlation coefficients (black: OFF, red: ON). We compared these to a surrogate distribution generated under the assumption of no systematic change. Trial labels were shuffled, reassigned across 25 FQ+N trials, and correlation coefficients were computed per participant and averaged. This process was repeated 1000 times. The observed correlations exceeded the surrogate confidence intervals (two-sided 95% CI; *P*_ON_ = .00003, *P*_OFF_ = .0103).


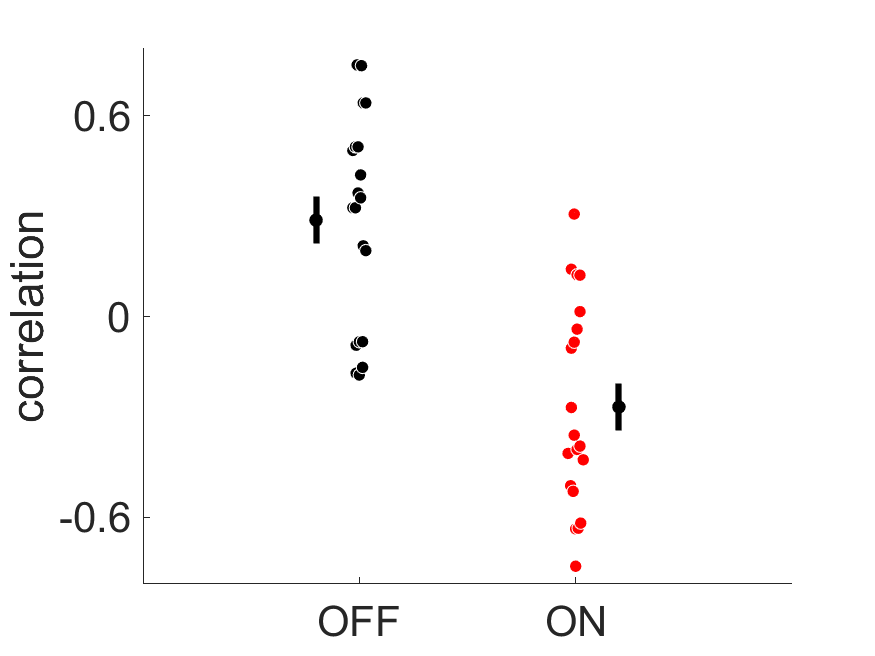


***Figure S2***. Depiction of how ON and OFF ratings changed over time after participants reported to be ON task. ON-task thought probes were identified and subsequent thought probe ratings were grouped by their temporal distance from these reports, resulting in mean ratings for each participant based on trial intervals (N trials) after an ON-task report. Available trial intervals were correlated with thought probe ratings using Spearman’s correlation, separately for ON and OFF. ON-task ratings (red) decreased, while OFF-task ratings (black) increased over time. Colored circles represent single data points.

***S3-Behavioral Consistency Between Observed and Predicted Labels Suggests Accurate Trial Classification***

We conducted several analyses to assess whether trials were systematically mislabeled. Our rationale was that labeling errors are minimal if behavioral differences observed with the original trial labels can be reliably reproduced using the predicted trial labels. Our first step to minimize a label error was choosing a frequency band that reflects individual ratios of ON and OFF ratings. We also compared behavioral performance. Our results show that the observed differences in performance, reaction times, and reaction time variability with the original labels are also present when using the predicted labels. Furthermore, we find that the temporal dynamics of the drift from ON to OFF can be captured both with the observed labels alone and with the predicted labels. These findings detailed below suggest that systematic mislabeling of trials is minimal.

First, we selected the frequency band that best reflects individual ratings. We then examined whether differences in mean performance between ON, MID, and OFF trials were also reflected in the predicted labels. While previous analyses did not demonstrate this effect for reaction times, we now address this gap. We focused on trials with thought probes, using their observed labels. Trials were grouped by ON, MID, and OFF labels, and mean reaction times were computed for each participant and label. The same procedure was applied to the predicted labels. Reaction times were longer in OFF trials (observed: 596.2 ms; predicted: 595.9 ms) compared to MID (observed: 507.8 ms; predicted: 505.6 ms) and ON trials (observed: 482.1 ms; predicted: 491.5 ms). Directed t-tests showed a significant difference between ON and OFF trials for both observed (*t*_19_ = 1.91, *P* = .0358) and predicted labels (*t*_19_ = 2.04, *P* = .028). No significant differences were found between observed and predicted ON trials (*t*_19_ = 0.65, *P* = .518) or OFF trials (*t*_19_ = -1, *P* = .33).

We further examined reaction time variability. Variability was higher in OFF trials (observed: 14.15; predicted: 14.17) compared to MID (observed: 4.6 ms; predicted: 4.61 ms) and ON trials (observed: 3.81 ms; predicted: 5.3 ms). Again, ON-OFF differences were significant for both observed (*t*_19_ = 2.005, *P* = .0301) and predicted labels (*t*_19_ = 2.007, *P* = .03), with no differences between observed and predicted ON variance (*t*_19_ = 1.007, *P* = .3264) or OFF variance (*t*_19_ = -1, *P* = .33;). These results indicate that reaction time patterns in observed labels are well captured by the predicted labels.

In another analysis we tested whether the differences in reaction times between ON and OFF trials were also present when we randomly reassigned brain state labels to trials. In 1,000 runs, we pseudorandomly assigned ON and OFF labels to trials and computed the t-value for the difference in ON versus OFF likelihood in each run, resulting in a distribution of 1,000 surrogate t-values. The observed t-value was then compared to this surrogate distribution. The p-value relative to the surrogate distribution was .017 for reaction times and .016 for reaction time variability, indicating that significant differences emerge only when using the predicted labels—not when these labels are randomly reassigned. These findings suggest that trials were not mislabeled.


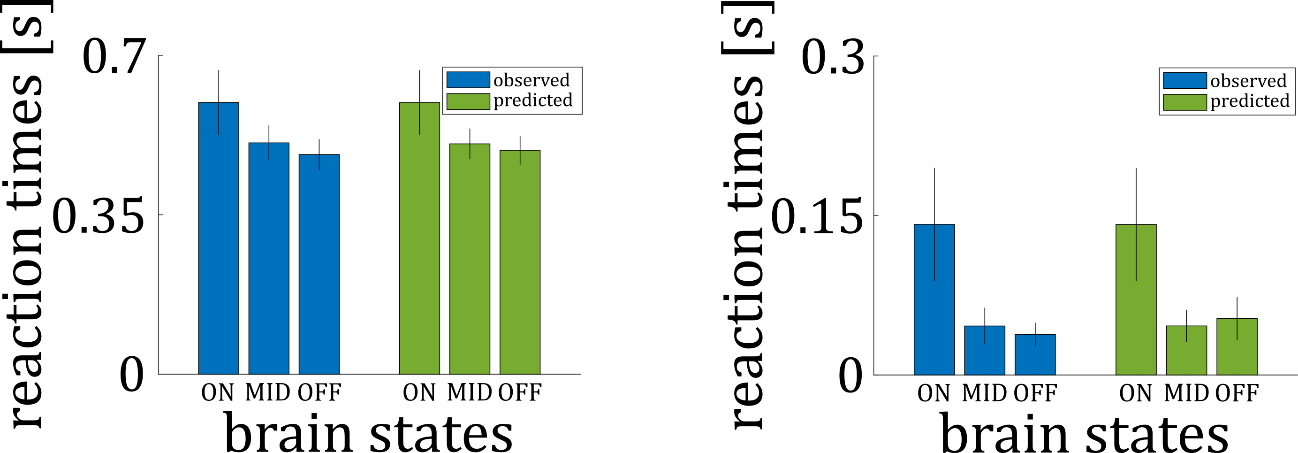


***Figure S3***. Brain state (ON, MID, OFF) reaction times (left) and reaction time variability (right) for observed and predicted labels. Reaction times and reaction time variability differed between brain states, but not between label types. The results show that reaction time patterns in observed labels are well captured by the predicted labels.

***S4-Potential influence of target eccentricity on brain states***

Higher target eccentricity (distance between target stimulus and fixation cross) can be associated with increased task difficulty and different neural processing demands. If participants reported mind wandering more frequently during more difficult trials (i.e., trials with more eccentric target positions), then observed neural differences between ON and OFF states could in principle be driven by external stimulus factors rather than internal attentional states. Thus, we analyzed whether target eccentricity differed between brain states. The 18 possible target positions were grouped into five distance levels based on their eccentricity relative to fixation. The two positions closest to fixation (upper right in the left matrix and upper left in the right matrix) were assigned to distance level 1. The two positions farthest from fixation (lower left in the left matrix and lower right in the right matrix) were assigned to distance level 5. Intermediate positions were assigned accordingly to distance levels 2–4.

The assumption is, if target position systematically influenced mind wandering reports (e.g., ON ratings predominantly for near targets and OFF ratings for far targets), we would expect ON ratings to be associated with lower distance and OFF ratings with higher distance. In contrast, if mind wandering reports were independent of target eccentricity, the expected distance level should approximate 3, reflecting an equal distribution across the five distance categories (i.e., 15/5 = 3). Hence, we examined the mean distance level of targets for each mind wandering rating.

We examined this question in two complementary ways: (1) based on the actually reported mind wandering ratings and (2) based on the classifier-derived state predictions.

In neither case did we observe a systematic deviation from the expected mean distance level of 3 (all p-values > .64). Thus, neither reported nor predicted ON and OFF states were associated with systematically different target eccentricities.

Moreover, the distribution of target positions did not differ across the different mind wandering levels. Together, these results indicate that target eccentricity was independent of attentional state and therefore cannot account for the observed differences in neural responses between ON and OFF conditions.


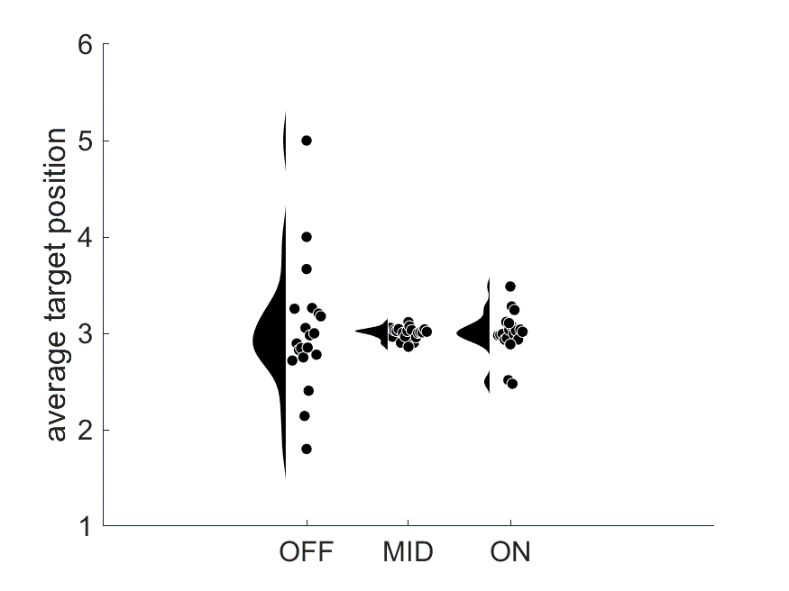

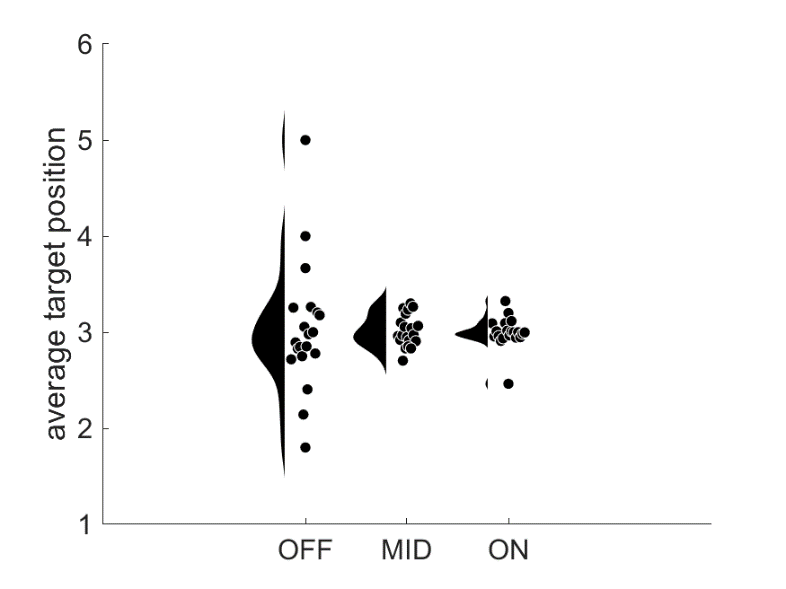


***Figure S4*:** Mind wandering ratings as a function of target position in the search display. The 18 target positions were grouped into five eccentricity-based distance levels (1 = closest to fixation; 5 = farthest; intermediate positions assigned to levels 2–4). Mean distance level did not differ across mind wandering ratings and was comparable for both reported and predicted labels, indicating no systematic relationship between target eccentricity and attentional state.

***S5-BHA variation with brain state***

We also analyzed possible confounds by mislabeled trials for the BHA. We first loaded the data and then assigned labels to the trials in a pseudorandom manner. Next, we grouped trials labeled 1 and 2 as "OFF" trials and trials labeled 4 and 5 as "ON" trials. This procedure was performed separately for each participant.

To assess differences between ON and OFF trials, we computed a t-value at each time point, repeating this process 1,000 times. As a result, we obtained 1,000 t-values for each time point, allowing us to estimate a 95% confidence interval (CI) for the difference between ON and OFF trials. The resulting confidence interval ranged from t_ci_ = [-1.75 1.75].

The observed t-values exceeded this confidence interval between 104 to 176 ms, with a maximum t-value of 2.47 occurring at 134 ms (*P* = .0097).

We further investigated whether BHA varied with ON and OFF state. In two separate steps we grouped only predicted labels and only observed labels into OFF (labels 1 – 2) and ON trials (labels 4 – 5) and averaged the baseline corrected time series across trials and stimulus-responsive MEG sensors in each subject.


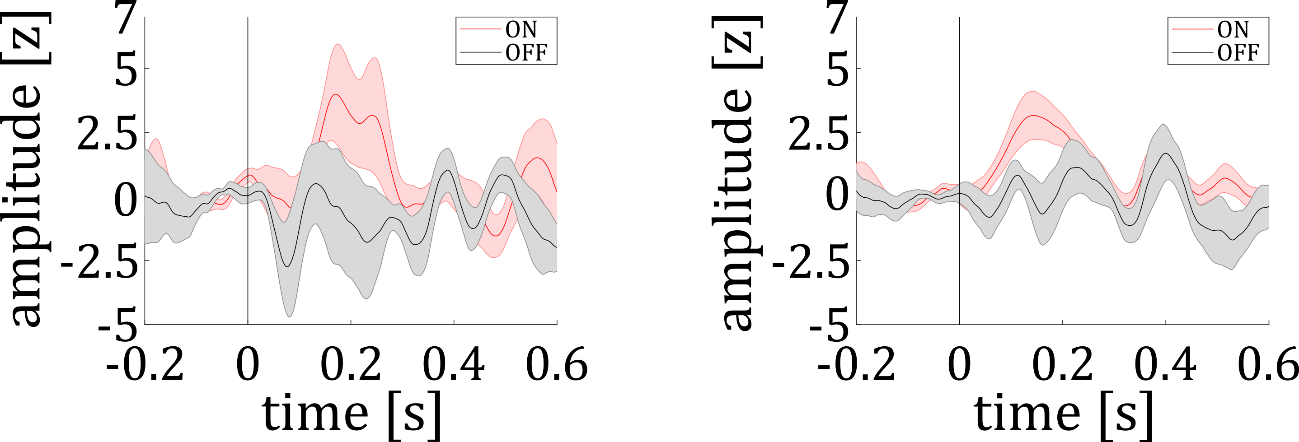


***Figure S5***. Time course of BHA for ON (red line) and OFF (black line) states for observed (left) and predicted (right) labels. Shaded colored lines represent the standard error of the means (SEM).

***S6-Comparison of BHA response characteristics between experiments***

In additional analyses we compared BHA response characteristics between the two experiments. Both responses show maximal amplitudes over occipital MEG sensors. Moreover, the responses do not differ in their peak frequency: a comparison of individual BHA peak frequencies revealed no significant difference between experiment 1 (mean peak = 131 Hz) and experiment 2 (mean peak = 123 Hz; *t*_41_ = 1.00, *P* = .32; see Figure S6). In contrast, BHA peak latency differed between experiments, with an earlier peak in experiment 2 (157 ms) compared to experiment 1 (247 ms; *t*_41_ = 3.94, *P* = .0003; ; see Figure S6). This difference is likely related to task relevance. In experiment 1, BHA is elicited by task-relevant gratings (one of which serves as the target), whereas in experiment 2 the checkerboard stimulus eliciting the BHA is task-irrelevant. Importantly, the overall temporal profiles of the responses following stimulus onset are otherwise highly comparable. The earlier decline of the response to the task-irrelevant stimulus is consistent with mechanisms of selective attention: while the initial sensory response is largely stimulus-driven, subsequent processing of irrelevant stimuli is rapidly suppressed by top-down control, resulting in a faster decay of neural activity due to the absence of recurrent amplification typically associated with behaviorally relevant stimuli.


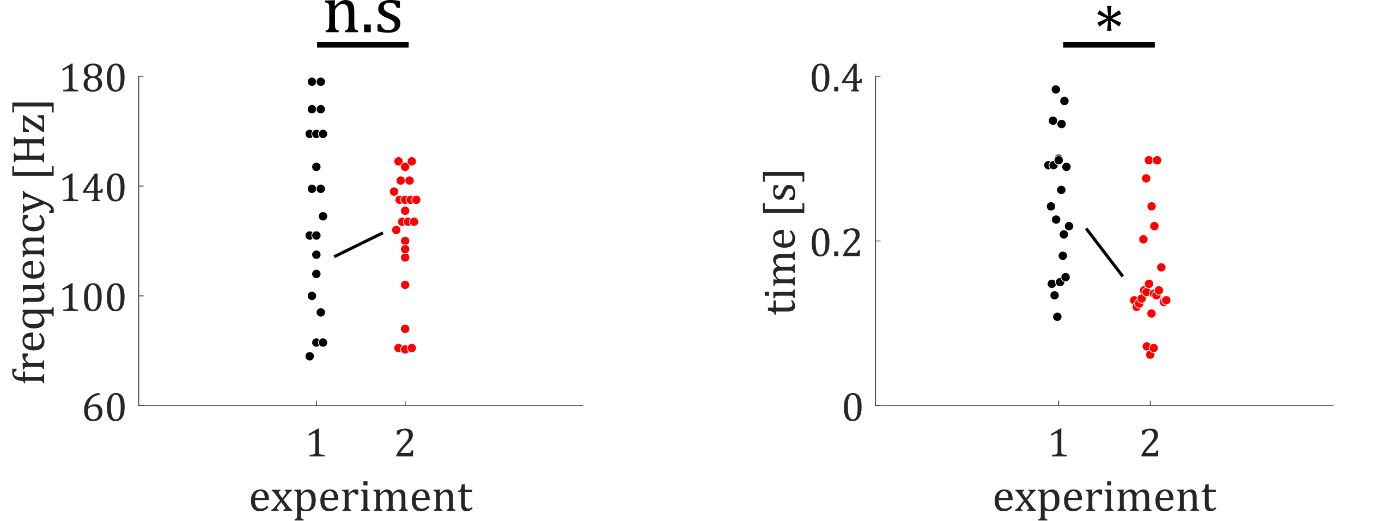


***Figure S6***. Individual BHA peak frequencies did not differ between experiments (left). Individual BHA peak latencies differed between experiments (right). Colored circles represent single data points. * = *P* < .001.
